# Why Some People Are Hesitant to Receive COVID-19 Boosters: A Systematic Review

**DOI:** 10.3390/tropicalmed8030159

**Published:** 2023-03-05

**Authors:** Yam B. Limbu, Bruce A. Huhmann

**Affiliations:** 1 Department of Marketing, Montclair State University, Montclair, NJ 07043, USA; 2 Department of Marketing, Virginia Commonwealth University, Richmond, VA 23284, USA

**Keywords:** vaccine hesitancy, booster, COVID-19, systematic review

## Abstract

As the COVID-19 pandemic continues and transitions to an endemic stage, booster vaccines will play an important role in personal and public health. However, convincing people to take boosters continues to be a key obstacle. This study systematically analyzed research that examined the predictors of COVID-19 booster vaccine hesitancy. A search of PubMed, Medline, CINAHL, Web of Science, and Scopus uncovered 42 eligible studies. Globally, the average COVID-19 booster vaccination hesitancy rate was 30.72%. Thirteen key factors influencing booster hesitancy emerged from the literature: demographics (gender, age, education, income, occupation, employment status, ethnicity, and marital status), geographical influences (country, region, and residency), adverse events, perceived benefit/efficacy, perceived susceptibility, perceived severity, prior history of COVID-19 infection, vaccination status, vaccination recommendations, health status, knowledge and information, skepticism/distrust/conspiracy theories, and vaccine type. Vaccine communication campaigns and interventions for COVID boosters should focus on factors influencing booster confidence, complacency, and convenience.

## 1. Introduction

The World Health Organization has received data on over 660 million confirmed cases of COVID-19, including over 6.7 million deaths across the globe [1]. Despite the availability of clinically tested and effective COVID-19 booster vaccines, convincing people to accept boosters remains a significant challenge [2]. As of 18 January 2023, only 15.3% of the total population in the United States (U.S.) had received the bivalent COVID-19 booster vaccine [3]. In addition, previous research shows people in many other countries [4,5,6], especially in low-income countries, are hesitant to receive COVID-19 boosters, e.g., [7,8]. However, other researchers have found relatively low levels of COVID-19 booster vaccine hesitancy (CBVH) in developed [9,10,11] and developing countries, e.g., [12,13]. These conflicting results highlight the need for a systematic review of CBVH.

In this study, booster hesitancy refers to delays in receiving boosters on the recommended timetable and refusal to receive boosters [14]. Booster hesitancy is not the inverse of booster acceptance (also known as uptake). People may receive a booster but postpone or delay the recommended schedule or accept some but not all recommended boosters due to hesitancy.

To successfully combat COVID-19, it will be necessary to overcome booster vaccine hesitancy. Refusals or delays in receiving recommended boosters threaten progress in tackling diseases [15,16]. Therefore, it is important to understand the factors driving refusals and delays.

Previous studies examined numerous factors that affected booster acceptance, including socioeconomic (e.g., occupation, education level, and income), demographic (e.g., gender, age, and ethnicity), geographical (e.g., urban/rural), psychological (e.g., self-efficacy, sense of control, and optimism), confidence-related (e.g., information source trust, vaccine effectiveness and safety concerns, and vaccine conspiracy beliefs) and experiential (e.g., prior COVID-19 infection, loss of peer/family to COVID-19, influenza vaccination status, and vaccine side effects) influences [17,18,19,20]. The strongest predictors of COVID-19 booster acceptance appear to be a history of chronic disease, trust in vaccine effectiveness, age older than 45, and the male gender [17]. Even with eventual acceptance, delays due to booster hesitancy can expose people to greater risks of infection. Further, all booster doses should be taken as recommended; only receiving one booster or just the primary vaccine series would expose people to COVID-19 as protective immunity wanes over time. Thus, research should separately identify factors influencing booster hesitancy.

Four systematic reviews have already been conducted on COVID-19 booster vaccines. However, two of these reviews focused on COVID-19 vaccine booster dose uptake and intention to get the booster dose [17,21]. A third systematic review by Chenchula et al. [22] reviewed the studies exploring the effectiveness of the booster or third COVID-19 vaccine dose against the new COVID-19 variant Omicron. A fourth study by Deng et al. [23] reviewed the studies to evaluate and compare the effectiveness and safety of heterologous booster doses with homologous booster doses for severe acute respiratory syndrome coronavirus 2 (SARS-CoV-2) vaccines. To the authors’ knowledge, no systematic review has been carried out to synthesize the literature on the determinants of COVID-19 booster vaccine hesitancy. The current review fills this critical gap to help researchers and policymakers better understand the factors influencing CBVH and develop interventions to reduce it.

The current study contributes to the literature by systematically reviewing the quantitative studies examining the factors influencing COVID-19 booster vaccination hesitancy. We will identify key themes of predictors and compare the consistency of the determinants’ influence to inform gaps in research and interventions to overcome hesitancy. Further, the results are broken down by the year of study, geographical region, and population type.

## 2. Methodology

This systematic review was carried out in line with the guidelines of the Preferred Reporting Items for Systematic Reviews and Meta-Analyses (PRISMA) [24].

### 2.1. Inclusion Criteria

The main inclusion criteria were quantitative empirical studies published in peer-reviewed journals and written in English that investigated the determinants of COVID-19 booster vaccine hesitancy (CBVH). We excluded the studies that examined the determinants of vaccination intention or acceptance. We did not include qualitative studies, conference proceedings, or grey literature.

### 2.2. Search Strategy

We comprehensively searched studies published from January 2020 to December 2022. We searched PubMed, CINAHL, Medline, Web of Science, and Scopus using various search terms, including booster, hesitancy, refusal, delay, reluctance, or unwillingness, and COVID-19, coronavirus, or SARS-CoV-2. As shown in Table 1, Boolean operators (AND, OR) were used to locate studies in the databases.

**Figure 1:**
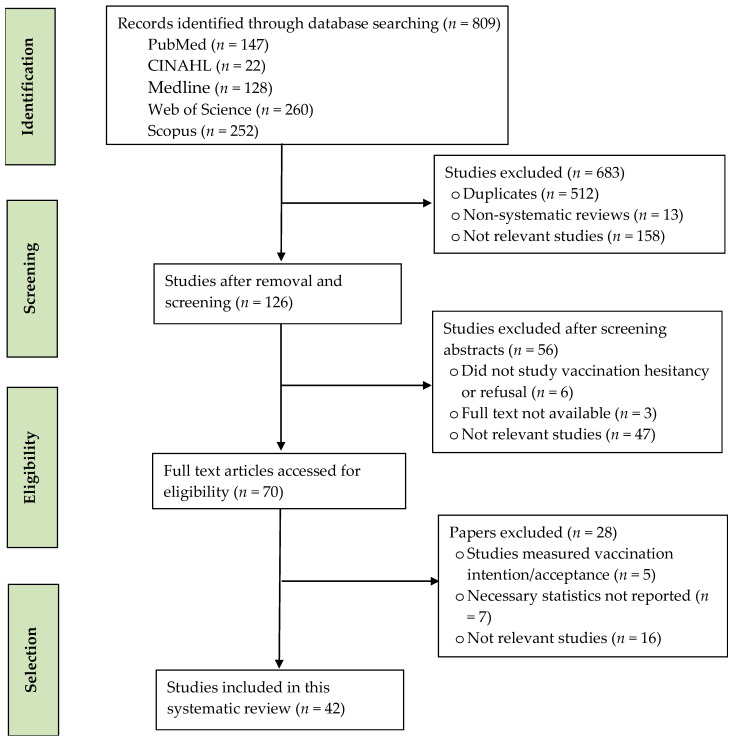
PRISMA flow diagram showing search strategy.

Two researchers independently screened all titles and abstracts of the identified articles, then assessed full texts in line with the inclusion and exclusion criteria described above. Figure 1 shows the summary of the article selection process according to the PRISMA guidelines. A total of 809 records were retrieved from the initial search on electronic databases. After removing duplicates and irrelevant studies, 126 records were retained for screening. Of these, 56 articles were excluded after screening the abstracts as irrelevant or not examining the predictors of COVID-19 booster vaccine hesitancy or refusal. A total of 70 articles were eligible for full-text screening. After removing those that did not measure hesitancy but instead measured another variable (e.g., intention or acceptance), only 42 studies were selected for this systematic review.

## 3. Results

### 3.1. Study Characteristics

Forty-two studies were included in this review representing 25 countries, the East Mediterranean Region (EMR), Latin America, and the Caribbean (see Table 2). Nine studies were conducted in China, followed by six in the U.S., three in Saudi Arabia, and two in India, Japan, Singapore, Poland, and Iraq. The majority of the studies (25/42) were conducted in Asia, followed by Europe (8 studies), North America (6 studies), Africa (3 studies), and South America (2 studies). No studies were conducted in Oceania. The vast majority of the studies (39/42) included in this review were published in 2022, and the remaining three in 2021. Two-thirds of the studies collected data in 2021. All studies were cross-sectional and employed survey methodology. Thirty-six studies (85.7%) used non-random sampling methods, and only six used random samples. Twenty-nine studies surveyed adults in the general public, five studies surveyed parents, four studies surveyed health care workers (HCW), and two studies surveyed patients. Participants in the remaining three studies were drawn from the student, army, and senior populations. The reviewed studies included 284,840 respondents, with an average sample size of 6927 respondents (standard deviation = 24,175.74), ranging from 224 [4] to 154,841 [25]. Only six studies (14%) used a theoretical framework such as the Health Belief Model, Protection Motivation Theory, and Theory of Planned Behavior. Thirty-one studies (73.81%) analyzed data using logistic regression analysis, fourteen (33.33%) used the chi-square test, and six (14.29%) used other statistical techniques.

### 3.2. COVID-19 Booster Vaccination Hesitancy Rate

Globally, the average COVID-19 booster vaccination hesitancy rate across all included studies was 30.72% (SD = 14.58), ranging from 2.1% among the general public of Japanese adults [52] to 56.63% among the general public of Croatian adults [33]. CBVH was highest in North America (41.18%), followed by Europe (34.81%), Asia (28.04%), and South America (27.6%). Vaccine hesitancy slightly increased from 2021 (29.46%) to 2022 (33.55%).

**Figure 2:**
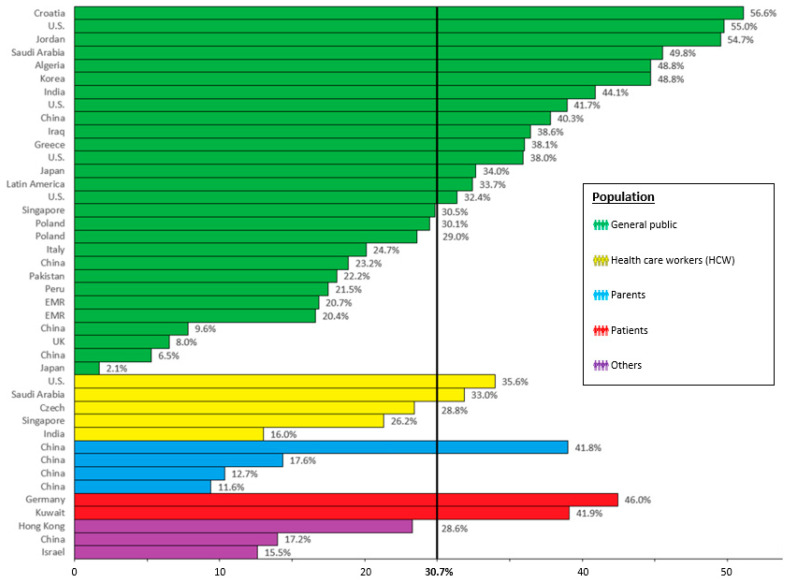
COVID-19 booster vaccine hesitancy (CBVH) rate by study population and geographical region compared to the average CBVH rate of 30.7% across all included studies.

Figure 2 shows CBVH rates for the reviewed studies organized by sample population. This figure also illustrates the range of CBVH rates across studies, with the greatest range in studies of the general public (2.1% to 56.6%). Hesitancy was the highest among patients (43.95%), with the least range in CBVH rates. The second highest average CBVH rate of 31.64% was found in studies of adults from the general public, followed by health care workers (30.2%). Interestingly, the average hesitancy was the lowest (20.92%) among parents considering COVID-19 booster vaccines for children.

### 3.3. Predictors of COVID-19 Booster Vaccine Hesitancy

#### 3.3.1. Demographic

As shown in Table 3, the most frequent demographic predictors of CBVH were gender, age, education, income, occupation, ethnicity, and marital status. Age was a significant predictor of CBVH in 16 studies (38%). Several studies reported that younger people were more likely to be reluctant to accept COVID-19 booster vaccines [2,4,5,6,8,10,12,25,29,32,36,37,45,52]. On the other hand, older individuals were associated with being less hesitant to get boosters [31].

Education attainment was the second most prevalent significant determinant of CBVH in 12 studies (28.6%). However, the results are inconsistent. While most studies revealed that a lower level of educational attainment was positively associated with a higher booster hesitancy rate [2,5,6,10,12,25,32,51], four studies revealed the opposite [5,8,41,49]. Another study found a positive relationship between a lower level of education and a lower CBVH rate [29]. Similarly, college students demonstrated a lower CBVH rate than other U.S. adults [31].

Gender predicted CBVH in 10 studies (23.8%). Several studies indicated that females are more likely to be hesitant to receive a COVID-19 booster vaccine than males [5,8,26,35,37,39]. On the contrary, Bendezu-Quispe et al. [12] found that females are associated with lower booster hesitancy. Moreover, two studies found that males are more hesitant [36,45].

Compared to other occupations, health care workers [8], allied health professions [37], housewives [41], administrative staff [38], and service workers [41] were more likely to be booster hesitant.

Evidence on the impact of income on CBVH is inconclusive. For example, three studies indicated that low income was associated with a higher CBVH rate [4,11,28]. Interestingly, in a study by Khan et al. [36], people with high household incomes and assets demonstrated lower CBVH. Moreover, Al-Qerem et al. [7] found that medium income was associated with higher CBVH.

Ethnicity was a significant predictor of CBVH in a few studies of U.S. populations. For example, a higher CBVH rate was associated with Blacks [31,39] and Native Americans [39], but Asian and Hispanic Americans had significantly lower CBVH rates compared to other races [31].

Employment status showed no clear association with CBVH across the reviewed studies. With respect to employment status, unemployed Chinese adults were more hesitant to receive boosters than their employed peers [11], but Bendezu-Quispe et al. [12] found just the opposite; employed adult Peruvians had a higher prevalence of not receiving the booster than unemployed adult Peruvians.

Consistent results were observed between CBVH and marital status. Compared to married adults, single or never married adults were more likely to be booster hesitant [2,51]. Conversely, married Japanese individuals were less likely to be booster dose hesitant than their unmarried peers [36].

#### 3.3.2. Geographical Factors

Geographical factors such as country, region, and residency were also associated with CBVH. Higher COVID-19 booster vaccine hesitancy was found among residents of Saudi Arabia [26], rural areas [12,28,31,39], and the southern U.S. [51]. Two studies reported greater CBVH among residents of towns than cities [12,25]. Interestingly, the study of some populations is geographically limited. Whereas several populations have been investigated in multiple geographic regions, all studies of parents to date have been conducted in China.

#### 3.3.3. Adverse Events

Vaccine-associated adverse events were the most frequently reported predictor of CBVH in 27 studies (64.29%). The main drivers for CBVH were fear about the side effects of booster vaccines [4,21,30,45,47,50], the severity of side effects associated with previous COVID-19 vaccines [26,29,31,34], concerns about adverse reactions to booster vaccines [6,52], adverse reactions experienced personally or among friends and family following previous COVID-19 vaccinations [5,7,30,52], and receipt of medical care following the COVID-19 vaccine primary doses [37]. Other studies reported uncertainty, risk, and safety concerns associated with booster doses [5,13,20,26,43,44,49,53].

#### 3.3.4. Perceived Benefits/Efficacy

Perceptions of the benefits and effectiveness of boosters were frequent determinants of CBVH. Greater CBVH rates were associated with a lack of confidence and trust in the effectiveness of the booster dose [2,4,8,30,31,47,51], concerns or uncertainty about the efficacy of COVID-19 vaccines [5,6,21,44,50,52], and low perceived benefits of boosters [34,43,49]. Similarly, high response efficacy (i.e., the belief that receiving a booster will prevent COVID-19) was significantly associated with lower hesitancy among Chinese adults [20].

#### 3.3.5. Perceived Susceptibility

Perceived susceptibility to COVID-19 was a significant predictor of CBVH. For example, booster vaccine hesitancy was strongly associated with low perceived susceptibility among Chinese adults [20,47] and people aged 60 years and older in China [43]. In addition, Iraqi adults who believed that they would not be infected with COVID-19 in the next six months [29], fully vaccinated adults in the U.K. who had lower stress about catching COVID-19 [10], and Singaporean adults who had a lower perceived risk of contracting COVID-19 [49] were more hesitant to receive a booster COVID-19 vaccination. Finally, Jordanian adults in the high-level group for developing COVID-19 complications had a lower hesitancy rate [7].

#### 3.3.6. Perceived Severity

Perceived severity (i.e., seriousness and consequences of contracting COVID-19) was a significant driver of CBVH in four studies. Lower perceived severity was associated with higher CBVH among Iraqi adults [29] and students in Hong Kong [40]. Similarly, Paul and Fancourt [10] showed that less anxiety about becoming seriously ill from COVID-19 was associated with greater CBVH in fully vaccinated adults in the U.K. However, Wu et al. [20] reported contradictory evidence that Chinese adults with higher levels of perceived severity were more hesitant to receive a booster.

#### 3.3.7. History of COVID-19 Infection

A previous history of COVID-19 infection was reported as a strong driver of CBVH in 11 studies (26.19%). Booster hesitancy increased with a history of prior COVID-19 infections [8,12,25,37], including infections after at least one dose [26,45]. In addition, hesitancy over boosters for one’s children was greater among parents with a history of COVID-19 infections [5]. Individuals unaware of COVID-19 infections in family or friends [9,28] or who did not personally know someone who had died due to COVID-19 [29] were more likely to be reluctant to receive booster doses. Conversely, one study found lower CBVH rates among those who knew someone who had been hospitalized or died of COVID-19 [31].

#### 3.3.8. Vaccination Status

Greater CBVH was exhibited by those who remained unvaccinated or were only partially vaccinated with the primary series of COVID-19 vaccines [2,13,27,28,37,51]. Similarly, individuals who had never received a vaccine against influenza were more unwilling to receive a booster COVID-19 dose [26,45]. In addition, parents who did not intend to vaccinate their children were more reluctant to receive booster doses [51].

#### 3.3.9. Vaccination Recommendations

Individuals who did not recommend COVID-19 vaccines to their family and friends [32] or did not receive vaccination recommendations from physicians, family members, or community workers [11] were less likely to receive COVID-19 boosters. Research also shows that booster hesitancy was low among people who received the primary series of COVID-19 vaccine due to imposed laws [7,29].

#### 3.3.10. Health Status

Previous research offers mixed evidence on the association between health status and CBVH. Several studies reported that people with poor self-rated health status [6], low self-rated health status after the second dose of COVID-19 vaccination [9], comorbidities [25], depressive symptoms [25], and vaccine allergies [27] had higher vaccine hesitancy. On the other hand, healthy individuals [10] with normal body mass index [26] and those having no immunosuppression [45] nor chronic diseases [25,45] were more hesitant to receive COVID-19 booster doses. Moreover, individuals living without vulnerable individuals at home [28], believing in natural immunity [13], having a higher antibody level [52], and possessing below-average cognitive function scores [48] were more likely to be hesitant. However, another study found lower vaccine hesitancy among those with good self-rated health status [36].

#### 3.3.11. Knowledge and Information

Research indicated that low levels of knowledge about COVID-19 [29] and its vaccine [11] are the main influences on CBVH. Vaccine hesitancy was higher in individuals with a lower level of vaccine literacy (i.e., a person’s ability to collect and understand reliable information about immunizations and use the acquired knowledge to make informed decisions to benefit their health) [2,51]. Luk et al. [40] examined the role of eHealth literacy (i.e., the ability to seek, find, understand, and appraise health information from electronic sources and apply knowledge gained to addressing or solving a health problem) and confirmed that young adults with lower eHealth literacy were more hesitant to receive COVID-19 booster vaccines. Other drivers of CBVH included concerns about not knowing enough about the vaccination [50], desire for additional information regarding the booster dose [9], not having received information regarding the booster dose from an official government organization [9], low subjective informativeness about SARS-CoV-2 [4], and believing that certain illnesses made them ineligible for vaccination [43].

#### 3.3.12. Skepticism/Distrust/Conspiracy Theory

Seven studies (16.67%) reported that COVID-19 booster hesitancy was higher among individuals who believed that boosters were unnecessary [21,26,35,43,44,49] or who were skeptical of the need for booster doses [42]. Distrust in the government, CDC, and health care system [31] also increased hesitancy. Individuals with less trust in governmental pandemic management [4] and COVID-19 vaccine information given by public health/government agencies [51] were more likely to be booster hesitant. Studies also showed that people who supported conspiracy theories [4] and those who believed that their health was in God’s hands [31] and COVID-19 was similar to seasonal flu [13] were more reluctant to receive boosters.

#### 3.3.13. Vaccine Type

Two studies reported that individuals who received Moderna primary doses were more unwilling to receive boosters [5,32]. However, another investigation showed that booster hesitancy was higher among those who received Pfizer vaccines [5]. Seboka et al. [13] and Shehab et al. [46] found higher booster hesitancy among people receiving the AstraZeneca vaccine. On the contrary, Al-Qerem et al. [29] reported lower booster hesitancy among AstraZeneca recipients. Collectively, these results on the effect of vaccine type on CBVH seemed largely inconclusive.

#### 3.3.14. Miscellaneous Determinants

Other factors that were associated with higher levels of booster hesitancy include political affiliation and leaning [2,31], noncompliance with COVID-19 safety guidelines [10,29], a lower level of behavioral confidence [2], a lower level of optimism [33], a myopic view [36], food insecurity [25], social networks as a vaccine information source [33], social influence [35], and unwillingness to pay for the booster [13]. However, higher levels of perceived self-efficacy [20], greater anxiety about the future [36], and perceived importance of social media [11] were associated with lower booster hesitancy.

## 4. Discussion

Boosters play an important role in providing longer and greater protection against infection, severe disease, and hospitalization. Hospitalization among adults 65 and older who had received the bivalent boosters authorized in the U.S. in the fall of 2022 was 84% lower than among unvaccinated adults and 73% lower than among vaccinated adults who had not received the booster [54]. Among all adults, emergency room or urgent care visits among those receiving the bivalent booster were 56% lower than unvaccinated adults, 50% lower than those who had received their last mRNA vaccination over a year ago, and 31% lower than those who had received their last mRNA vaccination 2–4 months ago [55]. These last results show the importance of reducing hesitancy, because delaying boosters longer than recommended can also negatively impact personal health. Despite the protective benefits of receiving timely boosters, studies included in this systematic review show that hesitancy is high even among fully vaccinated adults (e.g., >50% of Americans [2])

Thus, the primary objective of this study was to identify factors associated with CBVH in prior studies, themes in prior studies, and conflicting findings across studies to better direct ongoing research into efforts to counteract or overcome CBVH. This systematic review also weighs evidence uncovered by previous studies to identify factors that should be most effective in developing interventions to reduce CBVH. Prior research has shown that no single intervention can eliminate hesitancy [56,57]; thus, it was important for this systematic review to identify the most consistently influential determinants to help in the reduction of CBVH.

Geographically, the current study reviewed studies of CBVH conducted around the world. Studies on the topic were more prevalent with Chinese, other Asian, European, and U.S. populations. This does not indicate that CBVH is more prevalent in these countries but that research is more prevalent in these regions. Moreover, several studies included vaccine types that respondents had received. However, no consistent relationship was observed between vaccine type and hesitancy.

### 4.1. Demographic Factors

The reviewed studies frequently included demographic factors. However, their usefulness in explaining or influencing CBVH is questionable. One exception is age. Older adults were consistently less hesitant than younger adults. However, age more directly relates to the likelihood of suffering adverse consequences from a COVID-19 infection than most other demographic factors.

A couple of studies of U.S. populations found ethnicity or racial differences in CBVH [31,39], with results for Black and Asian Americans consistent with public health data on adverse consequences related to COVID-19 that records a lower rate of deaths per 100,000 for Asian, but a higher rate for Black, than White Americans [3]. However, CBVH was lower among Hispanics [31] despite a much higher rate of deaths per 100,000 than Asian or White Americans [3].

Findings across reviewed studies were inconsistent for gender, education, and income because these demographic factors have a less clear relationship with COVID-19 susceptibility. However, occupation was a surprisingly counterintuitive predictor with greater hesitancy rates among health care workers, who should have better knowledge of the benefits and risks of boosters, and service workers, who have a greater risk of COVID-19 exposure [8,38,41].

Thus, except for age, most demographic factors (e.g., gender or educational attainment) showed conflicting results across the reviewed studies. The contradiction in CBVH across studies found with demographic factors arises because they are not explanatory variables but carrier variables associated with predispositions, beliefs, or attitudes toward and access to vaccines and boosters [58]. Thus, future research needs to identify the underlying explanatory factors that determine CBVH associated with these demographic variables to help public health officials design more effective information campaigns and other interventions to reduce hesitancy. Once the underlying explanatory determinants associated with a demographic factor, such as age or ethnicity, are understood, the indirectly related demographic factors can serve to identify likely target audiences for booster information campaigns or specific interventions.

### 4.2. Booster Vaccine Hesitancy Model

The CBVH findings can be structured in terms of the three interrelated categories of influences—confidence, complacency, and convenience—that comprise MacDonald’s [14] model of vaccine hesitancy. Summarizing results from this systematic review into a similar booster vaccine hesitancy model, *booster confidence* would include determinants that build or undermine trust in booster safety and effectiveness, health care systems or health care workers who deliver boosters, and public health officials or government agencies who recommend or mandate boosters. For example, normative influences positively or negatively affect CBVH depending on the direction of the social pressure to adopt a particular booster-related belief or behavior to be accepted by a society or group. Normative influences that consistently reduced CBVH in the reviewed studies were formed through government mandates and booster recommendations from family or health care and community workers as well as by the internal consistency after one has encouraged others to receive a vaccination. However, normative influences also created social pressure that heightened CBVH when social networks or social media served as an important information source or one’s political affiliation or leanings predisposed one to accept political messages discouraging vaccinations or boosters. Thus, interventions that can harness normative social influence should be powerful. For example, information campaigns that rely on recommendations from celebrities, political figures, or groups trusted by the target audience should be more effective at reducing CBVH. Additionally, campaigns can explain how to overcome booster hesitancy among family and friends by providing information and strategies for effective normative influence on the booster hesitant from those close to them.

Studies in this systematic review also identified other factors that affected booster confidence. These factors consistently increased CBVH, such as beliefs in natural immunity, that boosters are unnecessary, that personal health is in God’s hands, and that COVID-19 is similar to seasonal flu. Hesitancy was also greater among those who were uninformed about or perceived a lack of sufficient knowledge about COVID-19 or the booster vaccines. Other factors that harmed booster confidence and increased CBVH were skepticism regarding the need for booster doses; support for conspiracy theories related to COVID-19 or the vaccinations against it; distrust in public health agencies and the health care system; and distrust in the government in general, its pandemic management, and the information it provided regarding COVID-19 vaccines.

*Booster complacency* increases booster hesitancy as self-efficacy (perceived or actual ability to take actions needed to receive a booster) decreases, as other health or life concerns outweigh the importance of receiving boosters, the less COVID-19 health risks seem compared to the risk of booster side effects, the more unessential boosters are believed to be in preventing COVID-19, and the more unconcerned people are about COVID-19.

Factors related to booster complacency that this review found to decrease CBVH include greater self-efficacy, self-ratings of good health, and not living with vulnerable individuals. Those that increase CBVH include the perceived susceptibility to and severity of COVID-19, the perceived benefits and efficacy of the boosters, and prior experience of and risk perceptions related to adverse events following primary vaccines or boosters, such as side effects. History of a prior COVID-19 infection increased hesitancy toward boosters for oneself or one’s children in several studies. Similarly, those without knowledge of COVID-19 infections or deaths among family or acquaintances also generally exhibited greater CBVH, although contradictory results were also present.

Booster complacency in terms of beliefs that boosters are unessential or that respondents were unconcerned with COVID-19 were evident in studies that found those who had not received any or full primary COVID-19 vaccines or flu vaccines and did not intend to vaccinate their children also exhibited greater CBVH. Booster complacency from COVID-19 being outweighed by other health concerns was also evident. Some studies showed that having other health issues or poor health led to greater CBVH.

*Booster convenience* affects hesitancy when barriers arise that increase the financial, social, time, mental, physical, or other costs of receiving a booster. For example, a belief that the vaccination process is complicated or wastes time, unwillingness to pay for the booster, and concerns about missing work to receive a booster were all associated with increased CBVH. The mental cost of understanding and navigating the booster vaccination procedure was evident in the association between below-average cognitive function scores and greater hesitancy. Although not directly measured, the distance to a vaccination site may have increased CBVH as higher rates were observed in geographical regions with lower population density (e.g., rural areas, Saudi Arabia, the southern U.S., and towns) than cities or other more populous areas.

Prior research suggests that interventions that target multiple factors are more effective [59]. Intuitively, targeting factors from different categories of the booster hesitancy model should make a greater reduction in CBVH. For example, interventions and information campaigns should be more successful if they target factors that affect booster complacency and convenience, booster confidence and complacency, or booster confidence and convenience rather than factors from only one category.

### 4.3. Limitations and Future Research

In addition to the future research agenda outlined above, some limitations of the current review underscore the need for future research into additional aspects of CBVH. First, while listing determinants that increase or decrease hesitancy is useful when research is sparse, a meta-analysis would better indicate the size and direction of the relationships and help to identify potential moderating variables and boundary conditions once sufficient research applying common measures and variables becomes available.

Second, many determinants of CBVH were identified in the literature, which may make identifying the few that most effectively combat hesitancy difficult. Moreover, a limitation of the systematic review methodology is its inability to compare the effectiveness of particular interventions statistically. Thus, future research should investigate which determinants are more effective and determine if this effect varies across geographic regions or cultures.

Another limitation is the exclusion of qualitative studies, which could provide important insights into booster hesitancy. However, we excluded qualitative studies because only a few had been published, and their wide range of methodologies would have further complicated the clarity of this systematic review’s results. However, as more qualitative studies of CBVH are conducted, a future review should examine this evidence for additional ways to design interventions and educational campaigns to combat CBVH.

Finally, this systematic review included only English-language articles indexed in the PubMed, CINAHL, Medline, Web of Science, and Scopus databases. A future review could expand the studies examined to include grey literature, studies published in languages other than English, and those not indexed in these five databases. This expanded set of studies may further support the influences on CBVH identified in the current review or expand the list of determinants.

## 5. Conclusions

This systematic review examined studies of CBVH to help identify important determinants of booster hesitancy. While demographic factors were measured more often, their role in influencing CBVH was generally inconsistent. Other factors (e.g., normative influence, prior experience or concerns with adverse events, severity and susceptibility perceptions, skepticism, and access) that may influence booster confidence, complacency, and convenience were shown to influence booster hesitancy more consistently.

## Figures and Tables

**Table 1:** Search strategy.

Database	Search Terms (Boolean Operators)	#Records
PubMed	(((((((((((booster[Title]) AND (hesitancy)) OR (refusal)) OR (delay)) OR (decline)) OR (reluctance)) OR (unwillingness)) AND (covid-19)) OR (coronavirus)) OR (2019-ncov)) OR (sars-cov-2)) OR (cov-19)	147
CINAHL	TI booster AND TX (hesitancy or refusal or delay or decline or reluctance or unwillingness) AND TX (covid-19 or coronavirus or 2019-ncov or sars-cov-2 or cov-19)	22
Medline	TI booster AND TX (hesitancy or refusal or delay or decline or reluctance or unwillingness) AND TX (covid-19 or coronavirus or 2019-ncov or sars-cov-2 or cov-19)	128
Web of Science	((((((((((TI=(booster)) AND ALL=(hesitancy)) OR ALL=(refusal)) OR ALL=(delay)) OR ALL=(decline)) OR ALL=(reluctance)) AND ALL=(unwillingness)) AND ALL=(covid-19)) OR ALL=(coronavirus)) OR ALL=(2019-ncov)) OR ALL=(sars-cov-2) OR ALL=(cov-19)	260
Scopus	Title (booster) AND ALL (hesitancy) OR (refusal) OR (delay) OR (decline) OR (reluctance) OR (unwillingness) AND ALL (covid-19) OR (coronavirus) OR (2019-ncov) OR (sars-cov-2) OR cov-19	252

**Table 2:** Study characteristics and factors influencing COVID-19 booster vaccination hesitancy.

Author(s)	Year of Publication	Data Collection Year	Country/Region	CBVH %	Population	Sample Size	Sampling Technique	Study Design	Predictors of CBVH
Abouzid et al. [26]	2022	2021	EMR nations of Egypt, Iraq, Palestine, Saudi Arabia Sudan	20.4	General public	3041	NR	C-S survey	Safety uncertainties, opinions that the booster dose is unnecessary, side effects associated with previous COVID-19 vaccines, history of COVID-19 infection, influenza-unvaccinated individuals, females, normal body mass index, country
Abullais et al. [27]	2022	2021	Saudi Arabia	49.8	General public	520	RS	C-S survey	Vaccine allergy, only a single dose of the COVID-19 vaccination, private hospital usage
Achrekar et al. [28]	2022	2021–2022	India	44.1	General public	687	NR	C-S survey	Unvaccinated with the primary series, low income, rural residents, not living with vulnerable individuals, family/friend history of COVID-19 infection
Al-Qerem et al. [7]	2022a	2021	Jordan	54.7	General public	915	NR	C-S survey	Income (medium), perceived susceptibility, post-vaccine symptoms, government mandate
Al-Qerem et al. [29]	2022b	2022	Iraq	38.6	General public	754	NR	C-S survey	Age (younger), education (low), low knowledge about COVID-19, did not know someone who had died due to COVID-19, low perceived susceptibility, low perceived severity, high perceived seriousness of COVID-19, low side effects from previous COVID-19 vaccination doses, received a COVID vaccine due to government mandate, low adherence to protective practices against COVID-19, vaccine brand (AstraZeneca vs. Sinopharm)
Babicki et al. [30]	2022	2021	Poland	30.1	General public	1528	NR	C-S survey	Lack of confidence in the effectiveness of the booster dose, experienced adverse events following vaccination, family/friend experienced adverse events following vaccination, fear of future complications
Batra et al. [2]	2022	2021	U.S.	41.7	General public	501	NR	C-S survey	Unvaccinated with the primary series, younger, single or never married, education (low), Republicans, low vaccine literacy, low vaccine confidence, low behavioral confidence
Bendezu-Quispe et al. [12]	2022	2022	Peru	21.5	General public	20,814	RS	C-S survey	Gender, age (under 75 years), education, employed, history of COVID-19 infection, living in a town or rural area
Bennett et al. [31]	2022	2021	U.S.	32.4	General public	3497	NR	C-S survey	Age; ethnicity; education; rural residents; non-Democrats; household member at least 60 years of age; knew someone who had been hospitalized or died of COVID-19; knew someone with severe vaccine side effects; belief that one’s health is in God’s hands; lack trust in the government, CDC, or health care system; lack trust in COVID-19 vaccine efficacy
Chrissian et al. [32]	2022	2021	U.S.	35.6	HCW	2491	NR	C-S survey	Age (younger), education (lower), Moderna vaccine recipient, missed work due to vaccine-related symptoms, would not recommend vaccine to family or friends
De Giorgio et al. [33]	2022	2021	Croatia	56.63	General public	1003	NR	C-S survey	Vaccine information source (social networks, general internet blogs/forums, and friends/acquaintances), low optimism about the future
Folcarelli et al. [9]	2022	2021	Italy	24.7	General public	615	NR	C-S survey	Need additional information regarding the booster dose, no friends or family diagnosed with COVID-19, not having received information regarding the booster dose from official government organization, lower self-rated health status
Galanis et al. [21]	2022	2022	Greece	38.1	General public	815	NR	C-S survey	Concerns about side effects, concerns about effectiveness, opinion that further vaccination is unnecessary
Ghazy et al. [34]	2022	2022	EMR	20.67	General public	2327	NR	C-S survey	Beliefs that booster doses have no benefit, severe side effects from prior doses
Huang et al. [35]	2022	2022	China	41.8	Parent	514	NR	C-S survey	Gender (mother), parental belief in need for booster, parental attitude toward booster, presence of people around parents hesitant about booster vaccines for children
Khan et al. [36]	2022	2022	Japan	34	General public	2912	NR	C-S survey	Gender (men), age (younger), subjective health status, future anxiety, marital status, have children, household income and assets, myopic (i.e., present-focused) view of future
Klugar et al. [37]	2021	2021	Czech Republic	28.8	HCW	3454	NR	C-S survey	Gender (female), pregnant, age (young), allied health professionals, prior infection, previously unvaccinated, previously sought medical care for COVID-19
Koh et al. [38]	2022	2021	Singapore	26.2	HCW	891	NR	C-S survey	First-dose hesitant, vaccine dose (booster vs. first dose), occupation (administrative staff vs. ancillary, medical, or nursing)
Kowalski et al. [4]	2022	2021	Germany	46	COVID-19 patients	224	NR	C-S survey	Age (younger), nationality (foreign), income (low), low trust in vaccination effectiveness, fear of negative vaccination side effects, low trust in the governmental pandemic management, low subjective informativeness about SARS-CoV-2, support of conspiracy theories
Lennon et al. [39]	2022	2021	U.S.	55	General public	12,887	NR	C-S survey	Gender (female), ethnicity (Black, Native American), residency (rural)
Lounis et al. [8]	2022	2022	Algeria	48.8	General public	787	NR	C-S survey	Gender (female), age (younger), HCW, education (high: postgraduate degrees), history of COVID-19 infection, regret after vaccine, belief vaccines are not efficient
Luk et al. [40]	2022	2021	Hong Kong	28.6	Students	290	NR	C-S survey	eHealth literacy, perceived danger of COVID-19
Ma et al. [41]	2022	2021	China	17.63	Parents	9424	NR	C-S survey	Education (higher), occupation (housewives vs. other occupations), occupation (service workers vs. other occupations)
Motta et al. [42]	2022	2022	U.S.		General public	1551	NR	C-S survey	Concerns about missing work for vaccination, unconvinced that additional boosters are necessary
Noh et al. [5]	2022	2021	Korea	48.8	General public and parents	2993	NR	C-S survey	*For self:* gender (women), age (younger), education (low), brand (Moderna or mRNA-1273), experienced serious adverse events following previous COVID-19 vaccination, concerns about safety, doubts about efficacy *For children:* parent with children aged < 18 years, younger, higher education, Pfizer (BNT162b2), history of COVID-19 infection
Paul and Fancourt [10]	2022	2021	U.K.	8	General public	22,139	NR	C-S survey	Healthy adults, perceived susceptibility, perceived severity, low compliance with COVID-19 government guidelines during periods of strict restrictions, education (lower), socio-economic (lower), age (lower: below 45 years)
Qin et al. [43]	2022a	2022	China	17.2	60 years or older	3321	RS	C-S survey	Belief that they were ineligible for vaccination due to certain illnesses, concerns about vaccine safety, belief that booster shots are unnecessary, limitation on their movements, low perceived susceptibility, perceived benefit (low to moderate), perceived barriers (moderate to high)
Qin et al. [44]	2022b	2021	China	11.56	Parents	1724	NR	C-S survey	Belief that vaccination process is complicated and time wasting, vaccine safety uncertainty, vaccine efficacy uncertainty, belief that booster is unnecessary
Rzymski et al. [45]	2021	2021	Poland	29	General public	2427	NR	C-S survey	Age (young), gender (male), absence of immunosuppression, no chronic disease, history of COVID-19 infection after 1st dose, never had influenza vaccination, prior booster side effects, fear of booster
Seboka et al. [13]	2022	2022	Pakistan	22.2	General public	787	NR	C-S survey	Unwillingness to pay for the booster, partial vaccination, safety concerns due to prior side effects of doses, belief that COVID-19 is similar to seasonal flu, belief in natural immunity, vaccine brand (AstraZeneca)
Shehab et al. [46]	2022	2022	Kuwait	41.9	Patients	499	NR	C-S survey	Vaccine type (AstraZeneca), biologic therapy
Sun et al. [47]	2022	2021	China	9.61	General public	1062	NR	C-S survey	low perceived risk of COVID-19 infection, worried about the rapid mutation of SARS-CoV-2, belief that the vaccine is ineffective, worried about side effects
Talmy and Nitzan [48]	2022	2021	Israel	15.5	Army	1157	NR	C-S survey	Below-average cognitive function score
Tan et al. [49]	2022	2021	Singapore	30.5	General public	1552	NR	C-S survey	Education (tertiary), lower COVID-19 threat perception, lower perceived benefits, higher perceived concerns, decreased need for booster vaccination, lower benefit/concerns differential score
Urrunaga-Pastor et al. [25]	2022	2022	Latin America and the Caribbean	33.7	General public	154,841	RS	C-S survey	Age (under 75 years), education (lower), having no or 1 to 2 comorbidities, town resident, has food insecurity, depressive symptoms, has had COVID-19
Vellappally et al. [50]	2022	2022	India and Saudi Arabia	16 and 33	HCW	530 and 303	NR	C-S survey	India: concerns about vaccine effectiveness, concerns about probable long-term side effects Saudi Arabia: concerns about not knowing enough about the vaccination, concerns about probable long-term side effects
Wang et al. [11]	2022	2021	China	6.5	General public	3119	RS	C-S survey	Unemployment, low monthly income, low scores of knowledge, low level of cues to action, low perceived importance of social media, official social media use, traditional media use
Wu et al. [20]	2022	2021	China	23.2	General public	8229	NR	C-S survey	High perceived severity, response cost (risk), low perceived susceptibility, low response efficacy (benefit), low self-efficacy
Yadete et al. [51]	2021	2021	U.S.	38	General public	2138	NR	C-S survey	Single or never married, less educated, lives in the southern region of the U.S., unvaccinated, did not intend to have their children vaccinated, very little to no trust in the COVID-19 vaccine information given by public health/government agencies, low vaccine confidence, low vaccine literacy
Yoshida et al. [52]	2022	2021	Japan	2.1	General public	2439	NR	C-S survey	Age (younger), concerns about adverse reactions, experience of adverse reactions, concern about the efficacy of the COVID-19 vaccine, higher antibody levels
Zhou, M. et al. [53]	2022	2021	China	12.7	Parents	1602	RS	C-S survey	Response cost/risk
Zhou, Y. et al. [6]	2022	2021	China	40.3	General public	1536	NR	C-S survey	Younger, less educated, less healthy, unsure of vaccines’ efficacy and adverse events

Note: CBVH = COVID-19 booster vaccine hesitancy, NR = non-random, RS = random sampling, C-S = cross-sectional. This table only lists predictors that significantly influenced CBVH.

**Table 3:** Predictors of COVID-19 booster vaccination hesitancy by themes.

Themes	Determinants of CBVH
Demographic	***Increases CBVH*** Age Younger [4,5,6,8,29,32,36,37,45,52] Younger: below 45 years of age [2,10] Under 75 years of age [12,25] Parent with children aged <18 years [5] Younger parent [5] Education Lower level [5,6,10,25,32,51] With some high school [2] Less than postgraduate [12] *Higher level* [8,41] *High: tertiary education* [49] *Parent with higher education* [5] Gender Females [5,8,26,37,39] Mothers [35] Pregnant women [37] *Males* [36,45] Occupation HCWs vs. other [8] Administrative staff vs. ancillary, medical, and nursing [38] Allied health professions vs. other HCWs [37] Housewives vs. those with other occupations [41] Service workers vs. those with other occupations [41] Income Low [4,10,11,28] Medium [7] *High household income among young adults* [36]
Demographic	Ethnicity Black [31,39] Native American [39] Employment Unemployed [11] *Employed* [12] Other demographic Marital status (single or never married) [2,51] Nationality (foreign) [4] ***Decreases CBVH*** Age Aged 65 and older [31] Household member is at least 60 years of age [31] Education College degree [31] *Low* [29] Gender Female [12] Income High household income and assets [36] Ethnicity Asian and Hispanic [31] Other demographic Marital status (married) [36] Having children [36]
Geographical	***Increases CBVH*** Country: (Saudi Arabia) [26] Living in a rural area [12,28,31,39] *Living in a town* [25] Living in a town compared to living in the city [12] Living in a southern region of the U.S. [51]
Adverse events	***Increases CBVH*** Fear of booster side effects [4,21,45,47] Severity of side effects associated with previous COVID-19 vaccine [26,34] Knowledge of someone whose vaccine side effects were severe [31] Safety concerns due to side effects after previous doses [13] Concerns about possible long-term side effects of booster vaccines [50] Fear of future complications of boosters [30] Concerns about adverse reactions to booster vaccines [6,52] Personal experience of adverse events following vaccination [30] Family or friend’s experience of adverse events following vaccination [30] Experience of adverse reactions such as nausea [52] Experience of serious adverse events following previous COVID-19 vaccination [5] Post-vaccine mild or no symptom [7] Receipt of medical care following the COVID-19 vaccine primer doses [37] Uncertainty about the vaccine safety [26,44] Concern about vaccine safety [5,43] Higher perceived concerns about boosters [49] High response cost (i.e., adverse effects of boosters may interfere with daily activities) [20,53] Missing work due to vaccine-related symptoms [32] ***Decreases CBVH*** Low side effects associated with previous COVID-19 vaccine [29]
Perceived benefit/efficacy	***Increases CBVH*** Lack of confidence in the effectiveness of the booster dose [2,30,51] Lack trust in the efficacy of the vaccine [4,31] Concerns about the efficacy of the vaccine [8,21,47,50,52] Uncertainty about COVID-19 vaccine’s efficacy [5,6,44] Beliefs that booster doses have no benefit [34] Lower perceived benefits of booster vaccines [49] Lower benefit/concerns differential score [49] Perceived benefit (low to moderate) [43] ***Decreases CBVH*** High response efficacy/benefit [20]
Perceived susceptibility	***Increases CBVH*** Adults who believed they would not be infected with COVID-19 in the next 6 months [29] Seniors aged 60 years and older with a low perceived susceptibility [43] Adults with a low perceived susceptibility [20,47] Fully vaccinated adults who felt less stress about catching COVID-19 [10] Adults who had lower perceived risk of contracting COVID-19 [49] ***Decreases CBVH*** Adults who were categorized in the high-level group of developing COVID-19 complications [7]
Perceived severity	***Increases CBVH*** Low perceived severity of COVID-19 [29,40] Low levels of current stress about becoming seriously ill from COVID-19 [10] *High perceived severity* [20]
History of COVID-19 infection	***Increases CBVH*** History of COVID-19 infection [8,12,25,37] History of COVID-19 infection after at least one dose [26,45] No family/friends tested positive for COVID-19 [9,28] Did not personally know someone who had died due to COVID-19 [29] Parents with history of COVID-19 infection [5] ***Decreases CBVH*** Knew someone who had been hospitalized or died of COVID-19 [31]
Vaccination status	***Increases CBVH*** Unvaccinated with the primary series [2,28,37,51] Partial vaccination with the primary series [13] People with a single COVID-19 vaccine dose vs. two or more doses [27] Never had influenza vaccination [26,45] Parents who were unwilling to vaccinate their children [51]
Booster vaccine recommendation or mandate	***Increases CBVH*** Would not recommend vaccine to family or friends [32] Low level of cues to action (i.e., vaccination recommendation from physicians, family members, or community workers) [11] ***Decreases CBVH*** Received primary series of COVID-19 vaccine due to imposed laws [7,29]
Medical conditions and health status	***Increases CBVH*** Poor self-rated health status [6] Lower self-rated health status after the second dose of COVID-19 vaccination [9] 1 to 2 comorbidities [25] Depressive symptoms [25] People with allergy to vaccine [27] *Good self-rated health status* [10] *Absence of immunosuppression* [45] *No chronic diseases* [25,45] *Normal body mass index* [26] Belief in natural immunity [13] Higher antibody levels [52] Not living with vulnerable individuals [28] Below-average cognitive function scores [48] ***Decreases CBVH*** Good self-rated health status [36] Higher anxiety about the future [36]
Knowledge/information	***Increases CBVH*** Low knowledge about COVID-19 [29] Low knowledge of COVID-19 vaccine [11] Low vaccine literacy [2,51] Low eHealth literacy [40] Concerns about not knowing enough about the vaccine [50] Want additional information regarding the booster dose [9] Did not receive information regarding the booster dose from an official government organization [9] Low subjective informativeness about SARS-CoV-2 [4] Belief that certain illnesses made them ineligible for vaccination [43]
Skepticism/distrust/conspiracy theories	***Increases CBVH*** Belief that the booster dose is unnecessary [21,26,35,43,44,49] Skeptical of the need for boosters [42] Distrust in the government, CDC, and health care system [31] Low trust in the governmental pandemic management [4] Very little to no trust in the COVID-19 vaccine information provided by public health or government agencies [51] Supports conspiracy theories [4] Belief that one’s health is in God’s hands [31] Belief that COVID-19 is similar to the seasonal flu [13]
Vaccine type and treatments	***Increases CBVH*** Moderna vs. Pfizer [32] Moderna vs. other vaccines [5] Pfizer vs. other vaccines [5] AstraZeneca vs. other vaccines [13,46] Biologic therapy (patients receiving vedolizumab) [46] ***Decreases CBVH*** AstraZeneca vs. Sinopharm [29]
Miscellaneous determinants	***Increases CBVH*** Political affiliation (Republicans) in the United States [2] Non-Democrats who did not vote for President Biden in the United States [31] Low level of adherence to protective practices against COVID-19 [29] Low compliance with COVID-19 government guidelines during periods of strict restrictions (e.g., lockdowns) [10] Low behavioral confidence (i.e., a lack of certainty in regard to receiving the vaccine booster) [2] Less optimistic about the future [33] More myopic view of the future (i.e., less concerned about future consequences) [36] Experiencing food insecurity [25] Perceived barriers (moderate to high) [43] Low level of cues to action [11] Worried about the rapid mutation of SARS-CoV-2 [47] Concerns about missing work to vaccinate [42] Vaccine information source (social networks, general internet blogs/forums, and friends/acquaintances) [33] Traditional media use [11] Social influence (presence of people around them hesitant about children receiving booster vaccines) [35] Vaccine dose (booster vs. first dose) [38] First dose-hesitant [38] Belief that the vaccination process is complicated and time wasting [44] Unwillingness to pay for the booster [13] Visitor of a private hospital [27] ***Decreases CBVH*** High perceived importance of social media [11] High self-efficacy [20]

Note: Determinants are divided into those that the included studies found to “Increase CBVH” or “Decrease CBVH”. Italicized text indicates reported findings that contradict the reported determinants in the majority of the included studies.

## Data Availability

Data generated in this study are available by contacting the first author, if requested reasonably.
